# The role of viruses in cancer development versus cancer therapy: An oncological perspective

**DOI:** 10.1002/cam4.5694

**Published:** 2023-03-07

**Authors:** Hossein Javid, Alireza Sharbaf Mashhad, Shaghayegh Yazdani, Mahsa Akbari Oryani, Sanaz Akbari, Nastaran Rezagholinejad, Mahboubeh Tajaldini, Mehdi Karimi‐Shahri

**Affiliations:** ^1^ Department of Clinical Biochemistry, Faculty of Medicine Mashhad University of Medical Sciences Mashhad Iran; ^2^ Department of Medical Laboratory Sciences Varastegan Institute for Medical Sciences Mashhad Iran; ^3^ Surgical Oncology Research Center Mashhad University of Medical Sciences Mashhad Iran; ^4^ Department of Medical Laboratory Sciences Mashhad University of Medical Sciences Mashhad Iran; ^5^ Department of Medical Laboratory Sciences Ilam University of Medical Sciences Ilam Iran; ^6^ Department of Pathology, School of Medicine Mashhad University of Medical Sciences Mashhad Iran; ^7^ Department of Biology Islamic Azad University Mashhad Iran; ^8^ Department of Biochemistry Payame Noor University (PNU) Mashhad Iran; ^9^ Ischemic Disorder Research Center Golestan University of Medical Sciences Gorgan Iran; ^10^ Department of Pathology, School of Medicine Gonabad University of Medical Sciences Gonabad Iran

**Keywords:** carcinogen, oncolytic viruses, treatment, virotherapy

## Abstract

Despite great medical advances, oncological research is still looking for novel therapeutic approaches due to the limitation of conventional therapeutic agents. Virotherapy is one of these new emerging therapeutic approaches that attract attention with their widespread applications. Virotherapy use lives oncolytic viruses or genetically engineered viruses that selectively infect the tumor cells, replicate, and disrupt the cancerous cells that also induce their anticancer activity by stimulating the host antitumor immune response. Moreover, viruses are widely used as target delivery vectors for specifically delivering different genes, therapeutic agents, and immune‐stimulating agents. In addition to having antitumor activity by themselves in combination with conventional therapeutic agents like immune therapy and chemotherapy, Virotherapy agents also elicit promising outcomes. Therefore, in addition to their promising result in monotherapy use, virotherapy agents can also be used in combination with conventional cancer therapy, epigenetic modulators, and even microRNAs without any cross‐resistance, which allows the patient not to be deprived of her routine medicine. Still, this combination therapy reduces the adverse effect of the conventional therapies. All together suggest that virotherapy agents as novel potential agents in the field of cancer therapy.

## Graphical abstract

Virotherapy agents as novel potential agents in the field of cancer therapy are one of the new emerging therapeutic approaches that attract attention in this field. One of the concerning factors is the role of viruses in cancer development, which needed to be explored against their anticancer applications.

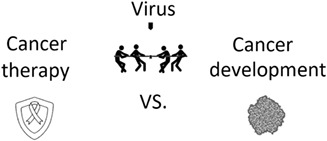

## 1 INTRODUCTION

Cancer is well known as a major global health concern.^1^, ^2^ The most conventional therapies that are already used for cancer and can extend the survival time of patients with cancer include surgery, radiotherapy, chemotherapy, and immunotherapy.^3^, ^4^, ^5^ However, all these conventional therapies have some limitations that have led to their failure in cancer treatment.

Surgery is most commonly used to remove the tumor in the early stages, but it cannot be an effective and sufficient treatment alone; hence, it is often combined with other cancer treatments, including chemotherapy and radiation.^3^ Furthermore, surgery and radiotherapy are ineffective in disseminated cancers and are more efficient against localized cancers; therefore, it seems that chemotherapy is the only choice.^3^ In addition, chemotherapy cannot be considered a sufficient therapeutic approach on its own due to the lack of specific toxicity for tumor cells. Moreover, in some cases, chemotherapeutic agents could lead to the development of multi‐drug resistant (MDR) cells.^6^ Another conventional cancer therapy is immunotherapy, which has only 10%–30% effectiveness.^7^, ^8^, ^9^ Therefore, there is an urgent need for new treatment strategies with potent tumor‐killing properties and fewer diverse effects.

Many viruses are effective in cancer treatment. Recently, virotherapy has attracted more attention as an effective agent in cancer treatment. Human intestinal cytopathic orphan viruses, adenoviruses, and herpes simplex viruses can replicate in tumor cells, causing cancer cells to die.^10^, ^11^, ^12^, ^13^ Besides, some virus species have anticancer effects by enhancing the host immune system.^14^ This study aimed to comprehensively review the role of viruses in the development of cancer as well as the latest advances in the anticancer applications of viruses.

## 2 VIRUSES IN CANCER DEVELOPMENT

**FIGURE 1:**
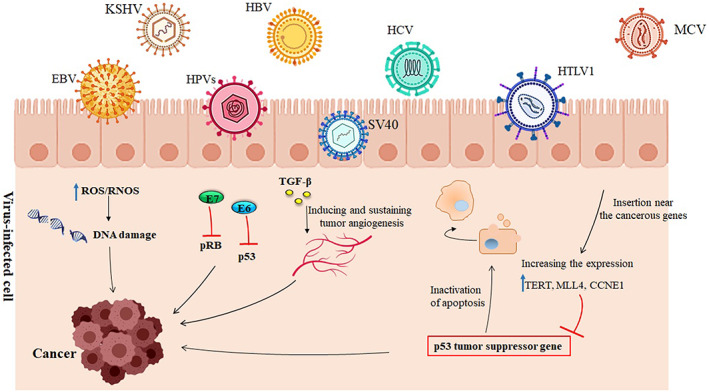
Oncogenesis mechanisms of viruses in cancer development. Viruses induce their carcinogen activity via insertion near the cancer genes such as telomerase reverse transcriptase (TERT), cyclin A2 (CCNA2), and cyclin E1 (CCNE1), and increase their expression, which results in inactivating the p53 and apoptosis mediated by that. Moreover, viruses, by increasing the expression of some factors like TGF‐β, can the tumor development due to the inducing and sustaining tumor angiogenesis. In addition, these viruses change and increase ROS production and induce oxidative stress (OS), resulting in DNA damage and then increasing the risk of tumor development.

Some studies suggest that viruses are the leading causes of nearly 10%–15% of all cancers worldwide. At the same time, other pieces of evidence claim that cancer development as a result of viral infections is usually a rare event.^15^ Although some viral infections can increase the risk of cancer, they do not necessarily cause the progression of cancer. According to epidemiological reports, the carcinogenesis of viruses depends on the virus load, the persistence of infection, and the duration of infection.^16^, ^17^ Some common viral carcinogenic features of cancer development include (i) direct transformation through the expression of viral genes, (ii) encoding oncoproteins, (iii) inactivating regulators of genome stability, (iv) interference in cell viability and cell cycle, (v) inactivating p53 and retinoblastoma proteins (pRB), (vi) activation of the DNA damage response, and (vii) changes to cellular levels of reactive oxygen species (ROS) and induction of oxidative stress (OS) (Table 1) (Figure 1).

**TABLE 1:** Basic features and associated cancers of oncogenic viruses.

Virus name	Genome	Family	Main target	Associated cancers	Anticancer mechanisms	Ref
DNA oncovirus						
Hepatitis B Virus (HBV)	Partially double‐stranded DNA (3.2 kb)	Hepadnaviridae	Hepatocytes	Hepatocellular carcinoma	Increasing the expression of TERT, MLL4, and CCNE1; Inactivating the p53; Disrupting in JAK/STAT and PI3K pathways; Upregulates the TGF‐β; Interfering in cell processes	24, 25, 26, 83
Human Papillomaviruses (HPVs)	circular, double‐stranded DNA (7.9 kb)	Papillomaviridae	Stratified squamous epithelium	Cervical cancer, penile cancers, head and neck cancers	Degradation of p53; Effecting on hTERT; Inactivating pRB; Increasing the generation of ROS and RNS; DNA damage	39, 40
Merkel Cell Polyomavirus (MCV)	Double‐stranded DNA (5.4 kb)	Polyomaviridae	Skin	Merkel cell carcinoma	Inactivating pRB; Suppress p53	^43^
Epstein–Barr Virus (EBV)	Double‐stranded DNA (172 kb)	Herpesviridae	Epithelium and B cells	Burkitt's lymphoma and nasopharyngeal carcinoma	Interfering in host genome; Effecting on BACH2	^47^
Kaposi's sarcoma herpesvirus (KSHV)	Double‐stranded DNA (165 kb)	Herpesviruses	Oropharyngeal epithelium	Kaposi's sarcoma, primary effusion lymphoma	Inactivating p53; Interfering in signaling pathways	^50^
Simian virus 40 (SV40)	closed circular double‐stranded DNA (5.2 kb)	Polyomaviridae	Human mesothelial cells (HM) and primary human astrocytes (Ast)	Brain tumors, osteosarcomas, malignant mesothelioma, and lymphomas	Inactivating p53; Disrupting pRb; Inactivate the RASSF1A; Activating the ERK‐kinase and AP‐1 pathways; DNA damage; Activating Met, Notch‐1, and IGF‐1R	55, 56
RNA oncoviruses						
Human T‐cell leukemia virus‐1 (HTLV‐1)	Positive‐strand, single‐stranded RNA (9.0 k)	Retrovirus	T and B cells	Adult T‐cell leukemia (ATL)	Surprising the hTERT; Interfering on NF‐κB pathway; Effecting p16, p15, Rb; ROS generation; DNA damage; Inactivating p53; Increasimg the CD4 + Foxp3+ Treg cells	180, 181
Hepatitis C virus (HCV)	Positive‐strand, single‐stranded RNA (9.6 k)	Flaviviridae	Hepatocytes	hepatocellular carcinoma (HCC) and lymphomas	Inactivating p53; Suppress p21WAF1; ROS generation; upregulating Bcl‐XL and Cyclin‐D; Inhibit apoptosis	75, 76

### 2.1 DNA oncoviruses

#### 2.1.1 Hepatitis B virus

Hepatitis B Virus (HBV) is well known as a hepadnavirus with double‐stranded DNA, which increases the risk of hepatocellular carcinoma (HCC).^18^ According to the reports, the HBV genome is observed in over 80% of HCCs, and there is a 5‐15‐fold increase in the incidence risk of HCC in people who are with chronic HBV carriers.^19^ Nevertheless, it is not always the case, as the integrated form of HBV is also detected in the non‐tumor tissues of people with chronic HBV infection.^20^, ^21^ However, genome integration of HBV into hepatocytes increases the risk of HCC development, which occurs during chronic infection of HBV and leads to an increase in the expression level of cancerous genes, including telomerase reverse transcriptase (TERT), mixed‐lineage leukemia 4, and encoding cyclin E1 (CCNE1) (encoding cyclin E1).^22^, ^23^

Overall, integrating the HBV genome raises cancer risk by causing additional genetic changes and interference with crucial cell processes. These processes include chromosomal deletions, translocations, the fusion of transcripts, DNA replication, and instability of the genome, which result in overexpression of oncogenes, repression of p53, inactivation of apoptosis mediated by p53, inactive cell cycle regulation, transactivation protein kinase C, JAK/STAT, and PI3K pathways, and upregulates the expression of TGF‐β.^24^, ^25^, ^26^, ^27^, ^28^

#### 2.1.2 Human papillomaviruses

Human papillomaviruses (HPVs) are double‐stranded DNA viruses that can infect epithelial cells.^29^ HPVs are well known as causative agents of the second most common cancer in women worldwide, called cervical cancer.^30^, ^31^ According to the reports, the DNA of HPV is observed in more than 90% of malignant squamous lesions of the uterine cervix. The HPV16, HPV18, HPV31, and HPV33 are the most common members of this family that are involved in more than 90% of all cervical cancer cases.^32^ However, HPV type 16 is considered the most diagnostic type, having been observed in more than 50% of all cervical cancer cases.^32^

In addition to cervical cancer, a high‐risk HPV infection can mediate other malignancies that account for more than 90% of anal cancers, 70% of vaginal and vulvar cancers, 60% of penile cancers, and 63% of oropharyngeal cancers.^33^ The E6 and E7 are the oncogenes encoded by HPV, which have a critical role in the cancer development process.^34^, ^35^ The integration of HPV‐16 into the host genome disrupts the E2 gene, which is a negative regulator for the expression of E6 and E7; hence, it leads to the high expression of these two oncoproteins and then cancer development.^36^, ^37^

The E6 oncogenes increase the risk of cancer by causing rapid degradation of p53, which is an important tumor‐suppressor protein that also activates human TERT (hTERT). Moreover, E7 also plays a role in cancer development via inactivating pRB, a tumor suppressor protein that prevents excessive cell growth.^38^ High generation of ROS and repetitive nerve stimulation (RNS) are the other factors that increase the integration risk of HPV through the further breaking of the DNA strand, which increases the integration of HPV‐DNA into cellular chromatin.^39^, ^40^ Furthermore, nitrative and oxidative DNA damage is also observed in the case of high‐risk HPV infections that play a role in cervical carcinogenesis mediated by inflammation.^41^

#### 2.1.3 Merkel cell polyomavirus

Merkel cell polyomavirus (MCV) belongs to the double‐stranded DNA polyomaviruses, which are well known as causative agents of Merkel cell carcinoma (MCC).^42^ The MCV induces its anticancer activity by encoding the tumor‐associated antigens and protein complexes that can target multiple tumor suppressor proteins, like pRB and p53.^43^ Recent findings have suggested that the association between MCV and MCC is very similar to cervical cancer induced by HPV due to the recurrent pattern of conserved viral DNA sequences, integration of MCV into the host genome, and expression of viral oncoproteins.^44^ However, more research is required to define the function of integration in MCC carcinogenesis.

#### 2.1.4 Epstein–Barr virus

Evidence suggests that Epstein–Barr virus (EBV), a member of double‐stranded DNA herpesviruses, is associated with several malignancies, such as Burkitt's lymphoma, nasopharyngeal carcinoma, and several lymphoproliferative disorders.^45^ In the case of Burkitt's lymphoma, there are three different clinical variants, including endemic, sporadic, and immunodeficiency, while EBV is observed in more than 96% of endemic variant Burkitt's lymphoma cases. In vitro studies have shown that EBV has transformative abilities through changing cellular gene transcription and activating cell signaling pathways, resulting in EBV‐encoded latent genes inducing B‐cell transformation into permanently latently infected lymphoblastic cell lines (LCLs).^46^ Unlike the others described above, the integration of the EBV genome with the host is rare. However, the integration of EBV into the fragile sites of the host genome causes partial deletion in the viral genome. Also, it generates a region that leads to instability in the host genome. This instability in the host genome leads to the loss of some host genes, like the BTB domain and CNC homolog 2 (BACH2), that are associated with tumor suppressor genes and are probably involved in lymphomagenesis.^47^ The available evidence suggests that EBV may play a role in nasopharyngeal carcinoma (NPC). These reports have investigated the integrated EBV in NPC biopsy samples and revealed the integrated EBV in some NPC cell lines that are EBV‐positive, HSB4, and H2B17–7.^48^ However, the role of EBV‐DNA integration and the risk of NPC is still unknown.

#### 2.1.5 Kaposi's sarcoma‐associated herpesvirus

Kaposi's sarcoma‐associated herpesvirus (KSHV) is a human gamma herpesvirus with a double‐stranded DNA herpesvirus. The KSHV is well known as the causative agent of primary effusion lymphoma and is common in AIDS patients.^49^ Moreover, this virus is associated with multicentric Castleman disease (MCD) and inflammatory cytokine syndrome. The anticancer activity of the KSHV is induced by encoded oncoproteins known as latency‐associated nuclear antigen 1, which inhibits the tumor‐suppressive activity of p53 and represses its transcription.^50^ Moreover, this oncovirus has anticancer activity via encoding the interferon regulatory factor‐like signal‐transduction protein, ORF K9, that blocks the signaling pathways induced by interferon. This inhibition protects oncoviruses from interferon‐associated antiviral function.^51^

#### 2.1.6 Simian virus 40

Simian virus 40 (SV40) is another oncogenic DNA virus associated with brain tumors, osteosarcomas, malignant mesothelioma, and lymphomas.^52^ This oncovirus has antitumor activity by encoding the large T antigen (LT), which can bind to the p53 gene, inactivate it, and inhibit the p53‐mediated cell death.^53^ Moreover, the LXCXE motif of this oncoprotein can bind to pRb, which results in the inactivation of the function of this tumor suppressor protein.^54^ In addition, LT is also able to inactivate the RASSF1A gene, which is a tumor suppressor gene.^55^, ^56^ Furthermore, LT of SV40 contributes to tumor development by activating the growth factor receptors, including Met, Notch‐1, and IGF‐1R, which enhance cell division and the carcinogenesis process by activating the extracellular‐signal‐regulated kinase and AP‐1 pathways.^57^, ^58^, ^59^ Moreover, this oncoprotein can induce the DNA damage response by binding to the mitotic spindle checkpoint kinase BUB‐1, which is in the best interest of the oncovirus.^60^, ^61^

### 2.2 RNA oncoviruses

#### 2.2.1 Human T‐cell leukemia virus‐1

Human T‐cell leukemia virus‐1 (HTLV‐1), well known as RNA oncovirus, belongs to the family Retroviridae and the genus Delta retrovirus and is associated with fatal T‐cell leukemia (adult T‐cell leukemia) and progressive myelopathy (HTLV‐1‐associated myelopathy/tropical spastic paraparesis HAM/TSP).^62^ HTLV‐1 promotes the proliferation of infected T cells by expressing Tax and HBZ, both of which have been linked to oncogenesis. The proliferation of infected T cells causes many infected T cells to have unique sites for integrating HTLV‐1 with the host genome. According to the reports, Tax has a role in tumor initiation, while HBZ is responsible for its maintenance.^63^ Tax is a 40‐kDa trans‐regulatory protein crucial in transforming infected cells into adult T‐cell leukemia. This oncoprotein can bind to the hTERT via occupied c‐Myc binding sites, resulting in unexpected hTERT expression. Tax is also capable of targeting the nuclear factor‐B (NF‐B) pathway, which is important in regulating antitumor immune responses. This oncoprotein also has anticancer activities by affecting proteins, such as p16, p15, and Rb, that are cell cycle inhibitors, and thereby leading to cyclins and cyclin‐dependent kinase activation. The tax also mediates the generation of ROS by affecting different pathways that result in DNA damage.^64^ Tax also has anticancer activity via inactivating the tumor suppressor protein p53. However, evidence suggests that tax is repressed after cancer development.

In contrast, HBZ is encoded in all adult T‐cell leukemia/lymphoma (ATLL) cells and HTLV‐1 infected cells, which are called ubiquitously expressed proteins.^65^ The HBZ is able to increase the CD4 + Foxp3 + Treg cells, which result in inflammatory disorders in several sites, such as the intestines, skin, and lungs.^66^ This protein also promotes the generation of infected cells by HTLV‐1.^67^, ^68^

#### 2.2.2 Hepatitis C virus

Hepatitis C virus (HCV) is a positive‐sense single‐stranded RNA virus and belongs to the family of Flaviviridae, which is also well known as the causative agent of HCC and human lymphomas. According to the reports, about 2%–3% (130–170 million) of people worldwide have been infected with HCV. There is an 11.5‐17‐fold increase in the risk of HCC development in people with HCV infection.^69^, ^70^ In other words, the incidence risk of HCC is about 15–30% within 20 years.^71^, ^72^ The HCV induces its oncolytic activity by encoding the oncoprotein called NS5A, which is able to bind to the p53 and suppress the transcription of p21WAF1.^44^, ^73^ Furthermore, NS5A can affect Bax and prevent apoptosis by binding to the p53.^74^ Moreover, this oncoprotein affects signal transduction, transcription, transformation, and ROS generation, resulting in the upregulation of Bcl‐XL and Cyclin‐D. All these allow HCV to induce chronic liver inflammation by changing the cytokine profile and disrupting the balance between apoptosis and proliferation which results in cancer development.^75^, ^76^

## 3 VIRUSES IN CANCER THERAPY

Virotherapy refers to using viruses to treat cancer that can find and destroy tumor cells specifically through different mechanisms without affecting normal cells. This method converts viruses into therapeutic agents by using biotechnology and reprogramming them to treat cancer. Virotherapy can be divided into three main groups, namely: (i) anticancer oncolytic viruses, (ii) viral vectors for gene therapy, and (iii) viral immunotherapy. All these approaches are based on therapeutic methods, including overexpression of the specific genes, usage of RNA methods to silence or decrease the expression of cancerous genes called gene knockout, and usage of the virus as a vector to deliver the gene that induces apoptosis and death in tumor cells, also known as “suicide gene delivery.”

### 3.1 Oncolytic virotherapy

These viruses induce their anticancer activity through rapid reproduction within the cancerous cells that leads to membrane ruptures and destruction, then the release of antigens that result in easy recognition and stimulation of the immune system to destroy the tumor cells.^77^, ^78^ Currently, oncolytic viruses attract more attention as therapeutic agents in the treatment of cancer (Table 2).

**TABLE 2:** Oncolytic viruses under clinical trials.

Virus family	Virus name	Description	Cancer	Clinical trial	ClinicalTrials. gov identifier
Adenoviridae	H101	A replication selective in tumor cells, recombinant, E1B and partial E3 gene deleted form of human adenovirus type 5, with potential antineoplastic activity.	Hepatocellular carcinoma	Phase III	NCT03780049
	DNX‐2440	Tumor‐selective; Expressing human OX40 ligand (OX40L, CD252, TNFSF4); Interactions in OX40 and OX40L; T‐cell activation	Colon cancer; Colorectal cancer; Breast cancer; Melanoma; Renal cell cancer; Squamous cell carcinoma	Phase I	NCT04714983
	DNX‐2401 (Delta‐24‐RGD)	Genetically engineered for replicates selectively in retinoblastoma (Rb) pathway deficient cells and tumor cells; elicits tumor necrosis; Triggers intratumoral immune cell infiltration; Can lead to long‐term patient benefit.	Glioblastoma	Phase I	NCT01956734
	TILT‐123	Engineered by E2F promoter and D24 deletion in the E1A gene; encode TNFa and IL‐2	Solid tumor; Melanoma	Phase I	NCT04695327
	VCN‐01	A replication selective in tumor cells; Encoding PH20 hyaluronidase; decreases intratumor fluid pressure; Increase the antibody uptake	Solid tumors; Pancreatic adenocarcinoma	Phase I	NCT02045602
	LOAd703	An adenovirus armed with CD40L and 4‐1BBL; activates CD40 and 4‐1BB pathways; Decrease the tumor growth factors (including Spp‐1, Gal‐3, HGF, TGFβ, and collagen type I); Increase cytokines and chemokines, and such DCs potently expanded both antigen‐specific T cells and NK cells.	Pancreatic adenocarcinoma; Ovarian cancer; Biliary carcinoma; Colorectal Cancer	Phase I/II	NCT03225989
	ICOVIR‐5	Optimized oncolytic adenovirus with E2F promoter and the Delta24 mutation of E1a; Selectivity for cell with deregulated E2F‐RB pathway.	Melanoma	Phase I	NCT01864759
	CG0070	A replication selective in tumor cells; Direct tumor cell lysis; Expression of GM‐CSF	Non muscle invasive bladder cancer	Phase III	NCT04452591
	CAdVEC	A replication selective in tumor cells; Enhance endogenous T lymphocytes activity; Increase the adoptively transferred CAR T cells.	Bladder cancer; Head and neck squamous cell carcinoma; Breast cancer; colorectal cancer; Pancreatic cancer	Phase I	NCT03740256
Herpesviridae	Talimogene laherparepvec	IMLYGIC is a genetically modified herpes simplex virus type 1; A replication selective in tumor cells; Inducing GM‐CSF and antitumor immune response; Ruptures tumors and then releases tumor‐derived antigens; Cell lysis.	Squamous Cell Carcinoma; Head and Neck Cancer, Melanoma, Pancreatic Cancer	Phase III	NCT01161498
	G47Δ	Inhibited tumor proliferation; Increased CD8+/CD45+ T cells G47D monotherapy inhibited the growth of the contralateral	Glioblastoma, Prostate cancer	Phase II	UMIN000015995
	rQNestin34.5	Induce dephosphorylated eIF‐2α in human glioma cells results in higher cytotoxicity; A replication selective in tumor cells; Elicits a tumor‐specific systemic immune and CTL responses.	Glioma; Astrocytoma; Glioblastoma	Phase I	NCT03152318
	G207	A replication selective in tumor cells; Induce CTL responses	Glioma; Astrocytoma; Glioblastoma;	Phase I/II	NCT00028158
Reoviridae	Pelareorep (Reolysin)	A replication selective in tumor cells; Activated Ras pathways; Stimulate NK cells and chemokines/cytokines responses; Induce T‐cells responses	Carcinoma, Squamous Cell of the head and neck, Ovarian cancer, Lung cancer	Phase II /III	NCT01166542 NCT01199263
Parvoviridae	ParvOryx (H‐1PV)	A replication selective in tumor cells; Cell cycle arrest at G2; Induce apoptosis; Dysregulation of cell transcription; Activation of cellular stress response; Induce cell death.	Glioblastoma, Pancreatic ductal carcinoma	Phase I/II	NCT01301430 NCT02653313
Paramyxoviridae	MV‐NIS	A replication selective in tumor cells; Tumor cell lysis; Induce tumor cell syncytia; Tumor‐specific killing.	Myeloma, Ovarian carcinoma, Peritoneal Carcinoma, Fallopian tube transitional cell carcinoma	Phase I/II	NCT02192775 NCT02068794 NCT00450814
Poxviridae	JX‐594 (Pexa‐Vec)	Lysis of tumor cells; Expresses GM‐CSF; Elicit the antitumor immune response.	Hepatocellular carcinoma, Melanoma	Phase II /III	NCT00554372 NCT01636284 NCT00429312

One of the most significant challenges in conventional cancer therapy is the lack of selective toxicity toward tumor cells with no side effects on normal cells. Viruses have been chosen as a new therapeutic approach to overcome this challenge due to their ability to target specific receptors that have overexpression on tumor cells that allow the selective entry of the virus. As an example, the measles virus can target the CD46 that has overexpression on multiple myeloma cells.^79^ Unbridled metabolism, as well as the rapid growth and division of tumor cells, make them a selective niche for many viruses, which is also advantageous for their replication, compared to non‐tumorous cells.^77^, ^78^ Furthermore, cancer cells mostly have alterations during the transformation process, such as losing the innate antiviral response pathways that make them susceptible to many more viruses compared to their non‐transformed cellular counterparts.

**FIGURE 2:**
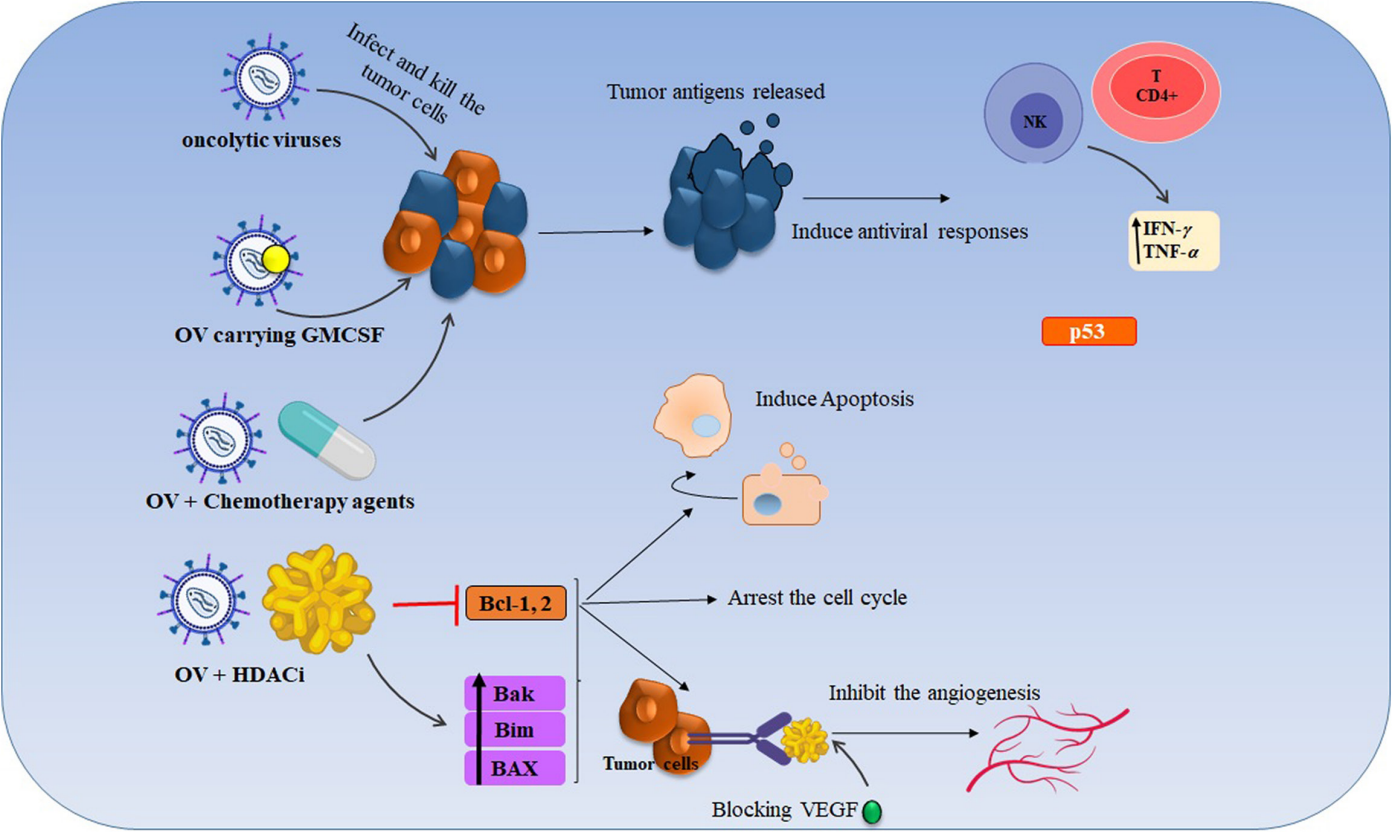
Anticancer mechanism of viruses. Oncolytic viruses (OV) induce their anticancer activity through different mechanisms like direct infection and killing the tumor cell or indirectly via stimulating and recruitment of the host immune system. Moreover, oncolytic viruses combine with other anticancer strategies such as chemotherapy and HDACi that enhance the anticancer activity by stimulating the T‐cell responses, inducing the apoptotic‐related genes (Bax, Bak, and Bim), arresting the tumor cell cycle, and inhibiting the angiogenesis. In addition, they are widely used as a delivery vector for gene therapy and immunotherapy agents that result in eliciting the antitumor immune responses.

Furthermore, cancer cells often are not able to induce antiviral responses, such as type I and II interferons (IFNs) or tumor necrosis factor (TNF).^80^ Newcastle disease virus (NDV) is well known as an RNA oncolytic virus that despite its cancerous nature exhibit anticancer activity and is able to selectively infects tumor cells.^81^, ^82^ Hepatitis B Virus (HBV) is another oncovirus that also exhibits anticancer activity by raising the expression of TERT, MLL4, and CCNE1, downregulating the intracellular level of tumor suppressor protein p53 and upregulating TGF‐β.^24^, ^25^, ^26^, ^83^ Oncolytic adenovirus is another example that probably induces cancer by hijacking the cell's crucial prosses and inserting its own nucleic acid into the host cells. However, this virus is also the same as the NDV despite its nature, widely used in cancer therapy, and mostly needs to be engineered and modified^84^, ^85^, ^86^ (more information about them is explained in title 3.2.). Some of them are engineered for targeting the specific receptors of tumor cells. Newcastle disease virus, autonomous parvovirus, and reovirus are non‐engineered viruses, while the adenovirus, herpes simplex, and vaccinia are some examples of the engineered viruses which have different abilities to lyse cells, activate the immune system, and transfer genes^87^, ^88^, ^89^ (Figure 2). The first oncolytic viral to be authorized by regulatory authorities for the therapy of cancer was RIGVIR, a non‐pathogenic intestinal cytopathic human orphan virus, which was approved in Latvia in 2004 for the treatment of melanoma. The modified adenovirus H101 (Oncorine, recombinant human adenovirus type 5 injection, ankeri), which was authorized in China in 2005, has not yet gained international recognition for its therapeutic efficacy.^90^ After that in 2015, T‐VEC for melanoma was the first oncolytic viral immunotherapy licensed by the US Food and Drug Administration (FDA) in 2015. T‐VEC (Imlygic®) is a modified herpes simplex virus (HSV) to produce the immune‐stimulating GM‐CSF protein in cancer cells and is less likely to infect healthy cells approved for certain subgroups of melanoma patients. It infects tumor cells and encourages their death. It is permitted for specific melanoma patients.^91^ The approval of T‐VEC for sale in Europe and Canada in 2016 signaled the development of oncolytic virus technology for the treatment of cancer. Other oncolytic virus products are undergoing phase III/II clinical studies^92^ (Table 2).

### 3.2 Viral vector in cancer therapy

As viruses have an immunogenic nature, researchers can engineer their genetic materials to change them into non‐infectious strains, and then use these recombinant viruses to carry any transgenes for their expression in tumor cells. Different recombinant viruses can deliver and express the transgene in immune cells, such as antigen‐presenting cells, most specifically dendritic cells, that stimulate the immune response toward tumor cells.^93^, ^94^ This manner is considered a type of gene therapy because recombinant viruses can alter genes in targeted cells. In addition to the cancer cells, these recombinant viruses can also target the malfunctioning cells which are involved in genetic diseases.^95^, ^96^ These recombinant viruses, which are also called viral vectors, can destroy the tumor cells directly or be used to stimulate tumor immune responses via the expression of tumor‐specific antigens^93^ (Figure 2).

#### 3.2.1 Viral gene therapy

Viral gene therapy is a radically new treatment that uses engineered viral vectors to deliver or introduce a foreign gene into the cancer cell. These modified viruses are used as delivery vehicles to introduce specific DNA sequences that encode genes and regulate RNAs (siRNAs), enzymes, antibodies, or other therapeutic substrates in the cancerous cells.^97^ Viral vectors are widely used as delivery vehicles of therapeutic agents due to their enhanced ability to permeate cells that are hard to access. All these features indicate that viral‐vector gene therapies can be considered a method for treating a wide spectrum of immunogenic diseases and cancer by controlling and programming specific gene expression.^98^ In addition to controlling the expression of specific genes, virally engineered vectors are also used to turn off the gene in diseases caused by overexpression. The three most common viruses are used as delivery vectors, including adeno‐associated virus vectors (AAV), adenovirus vectors, and lentivirus vectors.^99^ The AAV vectors, as well as adenovirus vectors, are mostly used as delivery vectors for gene therapy through direct administration to the host. However, lentivirus vectors are used for ex vivo therapies which means that the harvested host cells are modified in the laboratory before transplantation.^99^

#### 3.2.2 Viral immunotherapy

Viral immunotherapy is based on using viruses as delivery vehicles for immune‐stimulating substances, like tumor‐specific antigens, which help the host immune system recognize and fight tumor cells.^78^ Oncolytic adenoviruses are among the most prevalent viruses used as a vehicle for cancer immunotherapy. A cytokine transgene, granulocyte–macrophage colony‐stimulating factor (GMCSF), was recently added to the genome of an adenovirus, causing GMCSF production alongside virus replication. The production of GMCSF leads to the recruitment and maturation of dendritic cells that cause oncolysis and the induction of T‐cell responses by releasing the tumor‐specific antigens.^100^, ^101^ In addition to the genetically engineered viruses, the attenuated or killed virus is also used to generate an immune response in the host cells, which is well known as the vaccine.^102^ These vaccines are another branch of viral immunotherapy and are different from the vaccines that work against viruses. These vaccines do not prevent the disease but instead, affect the development of immunity. Vaccines that are used in cancer therapy are made of parts of cells or pure antigens which are tumor‐specific. Cancer vaccines are mostly used along with adjuvants which are well known as substances or cells that can help boost further immune response.^103^ Sipuleucel‐T (Provenge) is an example of a cancer vaccine used to treat advanced prostate cancer. Talimogene laherparepvec is another vaccine that has been approved to treat advanced melanoma skin cancer.^103^ Chimeric antigen receptor T cells are also considered as another form of viral immunotherapy.^104^ This strategy is based on using genetically engineered T cells that can produce an artificial T‐cell receptor that results in tumor cell killing. For this purpose, T cells will be transduced to the viral vector with a gene encoding the engineered chimeric antigen receptor after purification. To be safe, the most common viral vector used for this purpose is one created by integrating Gammaretrovirus into Lentiviral and having a partial deletion on their U3 region.^105^

## 4 VIRUSES IN CANCER DIAGNOSIS

Imaging technologies, like CT and MRI, play an irreplaceable role in cancer diagnosis; however, they are not effective in the primary identification of early stages of tumors and metastases. Currently, to overcome this challenge, oncolytic viruses are widely used to improve the efficacy of tumor imaging. The oncolytic viruses can selectively enter and replicate in tumor cells, carry specific genes, and express them into the tumor cells. Therefore, with the addition of genes, such as the luciferase reporter gene and human sodium iodine symporter gene, they can be detectable via gene expression products, such as fluorescence.^106^ Fluorescence imaging is one of the applications of oncolytic viruses in the field of the cancer diagnosis, which has higher accuracy and agility compared with the conventional diagnostic methods.^106^

Furthermore, the oncolytic viruses in the field of nuclear medical imaging have also attracted attention due to the reporter genes expressed in the tumor cells via these viruses that can acquire the exact location of tumor sites.^107^ The most common reporter genes are the human sodium iodine symporter gene, the thymidine kinase gene, and human type 2 somatostatin receptor gene. These viruses are detectable by different techniques, such as optical molecular imaging, bioluminescence imaging, and fluorescence imaging, single‐photon emission computerized tomography (SPECT) scanning, positron emission tomography scan, and magnetic resonance imaging. All this evidence declares that using the oncolytic viruses in combination with conventional imaging methods increases the chance of tumor diagnosis in the early stages.^107^, ^108^

## 5 VIRUS IN COMBINATION THERAPY

### 5.1 Viruses in combination with conventional cancer therapy

There are few but positive reports about using virotherapy along with conventional therapies for cancer^109^, ^110^, ^111^, ^112^ (Table 3). Radiotherapy is one of the most commonly used cancer treatments, and it has a synergistic effect when combined with virotherapy agents such as oncolytic Herpes simplex virus (HSV).^113^, ^114^, ^115^ This synergistic effect is due to the GADD34 induced by radiation which can enhance viral promoters via p38 followed by oncolytic HSV, leading to the blockage of the DNA repair.^116^, ^117^ In addition to the HSV, oncolytic vaccinia viruses can also improve the efficacy of radiotherapy through the inhibition of c‐Jun N‐terminal kinase signals. Furthermore, according to the reports, the vaccinia virus‐scAb‐vascular endothelial growth factor can improve radiotherapy efficacy by increasing tumor site sensitivity to radiation agents.^118^, ^119^ In addition to radiotherapy, chemotherapy is another conventional therapy that is used in combination with virotherapy. Cisplatin, 5‐fluorouracil (5‐FU), doxorubicin, temozolomide, irinotecan, and paclitaxel are some examples of chemotherapy agents used along with viruses for the treatment of cancer.^120^, ^121^, ^122^, ^123^ Different studies have revealed that this combination therapy, in addition to improving the antitumor effects, can also enhance safety and increase patient survival rates.^124^ The results of these studies demonstrate the synergistic effects of using the vaccinia virus along with paclitaxel, which can make cells enter the S phase of their cell cycle. That is the time the Vaccinia virus is more likely to infect cells.^125^ The preclinical result of the combination therapy of sorafenib with oncolytic vaccinia virus demonstrates promising antitumor effects.^126^

**TABLE 3:** Virotherapy agents in combination with chemotherapeutics

Virus family	Virus name	Drug	Cancer	Description	Clinical trial/Preclinical	Ref
Adenovirus	ONYX‐015 (CI‐1042)	Cisplatin, 5‐fluorouracil	Cervical carcinoma, Glioblastoma, Laryngeal carcinoma, Head and neck squamous cell carcinoma ovarian cancer	A recombinant adenovirus with a deletion in the E1B locus; kill cells with p53 mutations; A replication selective	Preclinical	^182^
	Ad‐ΔE1B19/55	Cisplatin	Cervical Cancer	An E1B‐mutant adenovirus; Selective replication in tumor cells; Induce apoptosis; Selectively lysis and killing the tumor cells.	Preclinical	^183^
	Telomelysin (OBP‐301)	pembrolizumab	Head and neck squamous cell carcinoma	Selectively replicate in tumor cells; Contains hTERT promotor; Elicit CD8+ T cell responses.	Phase I Phase II (NCT04685499)	^184^
	dl922‐947	Cisplatin, 5‐fluorouracil or gemcitabine	Malignant pleural mesothelioma (MPM), pancreatic cancer	Adenoviral E1A deletion mutant; replication‐selective in tumor cells; deregulating the cell cycle; killing cancer cells especially cells with K‐ras, p16, and p53 mutations.	Preclinical	185, 186
Poxviridae	MYXV	Cisplatin	Small cell lung cancer (SCLC), Ovarian cancer	Myxoma virus (MYXV) is a prototypic member of the Leporipoxvirus genus with the selective ability for infecting and killing the tumor cells; Induce tumor necrosis; increased infiltration of NK, CD8+ T, and CD45+ cells to the tumor microenvironment.	Preclinical	187, 188
Reovirus	Reolysin	paclitaxel and carboplatin	Squamous cell carcinoma of the head and neck	Selective replication in tumor cells; Activating Ras signaling pathway; activated PKR; lysis tumor cells specifically.	Phase III (NCT01166542)	^189^
Herpesviridae	G47Δ	Paclitaxel	Breast cancer	Inhibited tumor proliferation; Increased CD8+/CD45+ T cells	Preclinical	^190^

Furthermore, the results of the clinical trial of this combination therapy on cancerous patients have indicated an enhancement of the safety and clinical responses that also approve its systemic use in liver, kidney, and thyroid cancers. Besides, a few reports of using oncolytic virotherapy combined with immune checkpoint inhibitors, such as PD‐1 or/and CTLA‐4, result in immune response improvement.^127^, ^128^ All these findings indicated that virotherapy agents could enhance the antitumor effects of conventional cancer therapy; however, further studies are needed in this regard (Figure 2).

### 5.2 Viruses in combination with epigenetic modulators

Histone deacetylases (HDACs) are enzymes that remove the acetyl groups from ε‐N‐acetyl‐lysine residues on histones, resulting in histones tightly wrapping the DNA.^129^, ^130^, ^131^, ^132^ These enzymes are known as epigenetic modulators, which also have anticancer activity by arresting the cell cycle and inhibiting the proliferation of cancer cells.^133^, ^134^, ^135^, ^136^, ^137^ Suberoylanilide hydroxamic acid (SAHA) is an HDAC class I and II inhibitors with anticancer activity by inhibiting tumor cell proliferation, decreasing pro‐survival proteins (Bcr‐Abl, c‐raf, and protein kinase B), and upregulating cyclin‐dependent kinase inhibitor p21, resulting in cancer cell cycle arrest at the G1 phase.^138^, ^139^ The FDA has approved the SAHA as a pan‐HDAC inhibitor that also has anticancer activity by affecting apoptosis‐related proteins, such as blocking Bcl‐1 and Bcl‐2 and increasing Bim, Bak, and Bax proteins.^140^, ^141^, ^142^ Adenoviruses, combined with SAHA, have anticancer activity by arresting the cell cycle at the G2 phase, inducing apoptosis, increasing the tumor necrosis factor, and inhibiting the upregulation of p50 and p65 subunits of the nuclear factor kappa B (NF‐κB).^143^, ^144^ Rhabdoviridae is another oncolytic virotherapy agent used in combination with SAHA on prostate cancer cells. These two show antitumor activity by increasing the expression of NF‐κB target genes, decreasing the IFN, and inducing apoptosis.^145^, ^146^ Trichostatin A (TSA) is well known as a fungal antibiotic derived from *Streptomyces hygroscopicus* which also received FDA approval as a pan‐HDAC inhibitor. This antibiotic is also known as an epigenetic modulator because it is able to block HDACs classes I and II.

Furthermore, TSA inhibits breast and prostate cancer growth by arresting the cell cycle and regulating apoptosis‐associated proteins.^147^, ^148^ Combination therapy with Herpesvirus and TSA on glioma and colorectal cancer has shown that these two agents have antitumor and antiangiogenesis activities by blocking VEGF and Cyclin D1 degradation.^149^, ^150^, ^151^, ^152^ Furthermore, this combination therapy for oral squamous cell carcinoma shows anticancer activity through increasing the cytoplasmic NF‐κB activity.^149^ DNA methyltransferase (DNMTs) gene encodes the enzymes and is important in epigenetic regulation. Currently, it has been reported that DNA hypermethylation plays a fundamental role in cancer development.^153^, ^154^ DNA hypermethylation is widely reported in different types of cancer, including colon, breast, liver, bladder, ovarian, esophageal, prostate, and bone cancers.^155^, ^156^, ^157^, ^158^ Therefore, DNMT inhibitors (DNMTi) can be considered promising agents for cancer treatment. Azacitidine (5‐AZA) and decitabine (5‐aza‐20‐deoxycytidine) are two of the most common examples of DNMTi that have received FDA approval for usage as treatments for acute myeloid leukemia and myelodysplastic syndrome. These two are well‐known cytidine analogs that must be incorporated into the genome during the S phase in order to function. 5‐AZA can integrate with both DNA and RNA, whereas decitabine can only integrate with DNA.^159^ Different studies have used DNMTi in combination therapy for cancer with oncolytic viruses, which show strong stimulation of the immune responses as well as enhancement of the anticancer effects. Recent reports have demonstrated that combination therapy of oncolytic HSV‐1 with 5‐aza can synergistically induce apoptosis in glioma tumors, increasing the survival rate in mice bearing orthotopic human gliomas.^160^ According to one study, combining Rhabdoviridae with DNMTi increased anticancer activity and resulted in tumor remission in 70% of the cases^161^ (Table 4).

**TABLE 4:** Virotherapy agents in combination with epigenetic modulators which are in preclinical.

Virus family	Virus name	Epigenetic modulators	Cancer	Description	Result	Ref
Adenoviridae	ZD55‐Trial	SAHA	Cervical cancer	A replication selective in tumor cells, specifically target and lysis the tumor	ZD55 Harboring TRAIL and SAHA synergistically act to kill tumor cells; Inducing cell cycle arrest at G2; Inhibited tumor growth; Upregulation of IκBα, p50, and p65 subunits; Induce apoptosis	^143^
Reoviridae	Reolysin	SAHA and entinostat	Multiple myeloma (MM); Head and neck squamous cell carcinomas (HNSCC)	Activate Ras signaling pathways; Specifically kills tumor cells; Viability and replication in the hypoxia condition of tumor; cytopathic effect	Increased JAM‐1 and virus entry; Elicit the antitumor immune responses;	^191^
Rhabdoviridae	VSVD51	vorinostat; MS275; SIRT1; decitabine	Prostate cancer T‐cell lymphocytic leukemia	A replication selective in tumor cells; Decreasing the IFN‐responsive gene expression; Induce apoptosis	Induce apoptosis and autophagy; Enhance the oncolysis activity of virus; Increase NF‐κB activity	^192^
Parvoviridae	P/V‐CPI	scriptaid	Small cell lung cancer; Laryngeal carcinoma cells	Inhibit the IFN signaling; Targeting STAT1; Inhibit the expression of IFN‐β	Induce apoptosis; Suppressed IFN‐β; Inhibiting the phosphorylation and nuclear translocation of IRF‐3	^193^
	H1PV	VPA; NaB	Cervical cancer; Pancreatic cancer Carcinoma	Induces oxidative stress in tumor cells; Increase DNA damage; Arrest the cell cycle; Induce apoptosis	Increase the acetylation of the effector proteins of virus; Improve replication of virus; Increase oxidative stress	^194^
Paramyxoviridae	MeV	Resminostat	Hepatocellular carcinoma (HCC)	Induce production of IFN‐β; A replication selective in tumor cells	Stimulate the cellular innate immunity; Induce apoptosis; Enhance the replication of virus	^195^
Poxviridae	vaccinia viruses (VV)	Trichostatin A (TSA)	Colon carcinoma	Engineered VV with deletion of the B18R gene is more rapidly cleared from normal tissues while remaining active within tumors; Selectively growth in tumor cells with high levels of cellular thymidine kinase gene; Activated EGFR/Ras pathway signaling	Inhibit the IFN‐response; Enhance the replication of virus and its spread	^196^
Herpesviridae	G47Δ	Trichostatin A (TSA)	Glioma; Colorectal cancer	Inhibited tumor proliferation; Increased CD8+/CD45+ T cells G47D monotherapy inhibited the growth of the contralateral	Decrease VEGF and cyclin D1	^197^
	rQNestin34.5	VPA; TSA; NaB 5‐AZA	Glioblastoma Glioma	Induce dephosphorylated eIF‐2α in human glioma cells results in higher cytotoxicity; A replication selective in tumor cells; Elicits a tumor‐specific systemic immune and CTL responses.	Enhance the replication of virus; Increased IFN‐I expression; Induce tumor apoptosis	^160^
	R849	Trichostatin A (TSA)	Oral squamous cell carcinoma (SCC)	A neurovirulent with deficient in γ134.5 gene; Has LacZ genes at the deleted sites; Tumor suppressive effects.	Enhance the activity of NF‐kB; Improve viral production Raise the p21 interrupt; Arrest the cell cycle in G0/G1 phase	^149^
	BHV‐1	5‐AZA	Spontaneous breast fibrosarcomas	Selectively growth in tumor cells and specifically kill them	Induce apoptosis; Enhanced tumor cell clearance; Improve the infiltration of immune cells; Significantly decreased the incidence of secondary lesions	^198^

Abbreviations: IRF‐3, Interferon Regulatory Factor 3; JAM‐1, Junctional adhesion molecule 1; SAHA, suberoylanilide hydroxamic acid; TRAIL, tumor necrosis factor‐related apoptosis‐inducing ligand.

### 5.3 Viruses in combination with microRNAs

MicroRNAs (miRNAs) are well known as small non‐coding RNA molecules with regulatory roles affecting the expression of numerous gene networks at the post‐transcriptional level.^162^ Furthermore, the molecules with lengths of about 22 nucleotides are involved in various cellular functions, such as proliferation, metabolism, cell death, migration, and cell cycle; therefore, any dysregulation of miRNAs leads to tumorigenesis and cancer‐related processes.^163^, ^164^, ^165^ Current studies focus on miRNA‐based oncolytic virotherapy for cancer. In this manner, target sequences of miRNAs have been integrated into the genome of the virus, enhancing the safety profile of viral agents, and improving their anticancer efficacy by regulating the viral proteins. Several studies used the downregulation of specific miRNAs to improve the specificity of oncolytic virotherapy agents toward tumor cells and decrease their toxicity. For this purpose, synthetic target sequences complementary to specific miRNAs were inserted into the untranslated regions (UTRs) that are essential for viral replication. This method causes the degradation of the viral genome in normal tissues but not in cancerous tissues.^166^, ^167^, ^168^

In a study, a complementary target sequence to the miRNA‐145 was integrated into the 3’ UTR of the ICP27 gene that plays a role in encoding the glycoprotein of oncolytic HSV‐1. The aforementioned study showed that this insertion enhanced the selectivity of killing HSV‐1 for NSCLC tumor cells compared with normal cells.^169^ Another study demonstrated that combination therapy using miR‐122 as hepatic‐specific miRNAs and oncolytic adenovirus could significantly counteract hepatotoxicity and enhance the virus specificity for different types of cancer cells.^170^ The serotype 5 adenovirus (Ad5) is another oncolytic viral therapy for cancer that is used with miRNA. In this study, eight target sequences of the miR‐148a/miR‐152 family were inserted into the Ad5 genome downstream of the E1A gene. The result of this study demonstrated that this modification decreased the adenoviral infection in healthy pancreatic tissue, while enhancing the anticancer effect of the virus on pancreatic cancerous tissues.^171^ Coxsackievirus B3 is also modified with miRNA to increase tumor specificity.^172^ In a study, complementary target sequences of miR‐34a were inserted into the 30 UTR and 50 UTR of the coxsackievirus B3 genome which is called 53a‐CVB. This recombinant virus, with no toxicity for healthy tissues has strong anticancer activity in lung cancer cells^173^ (Table 5).

**TABLE 5:** Virotherapy agents in combination with microRNAs (miRNA).

Virus family	Virus name	miRNA	Cancer	Description	Result	Ref
Adenoviridae	AdD24.CMV‐GFP	miR‐26b	Prostate cancer	Oncolytic adenovirus carrying an expression cassette for enhanced GFP under control of the CMV promoter; A selective internalization to the tumor	Enhance the tumor proliferation; Decrease spread	^199^
	Ad‐L5‐8miR148aT	miR‐148a	Hepatocytes/liver	Engineered oncolytic adenovirus which containing eight miR‐148a binding sites downstream the L5 coding sequence; Specifically infect and kill the tumor cells	Improve the hepatotoxicity; Stimulate the anticancer response; Reduces fiber gene expression	^200^
	AdCN205‐IL‐24‐miR‐34a	miR‐34a	Hepatocellular carcinoma (HCC)	Engineered to express the higher level of miRNA‐34a and IL‐24; dCN205‐IL‐24‐miR‐34a significantly induced dramatic antitumor activity	Inhibited the expression of Bcl‐2 and SIRT1; Induce tumor regression without recurrence	^201^
Herpesviridae	dnU L 9‐T21	miR‐21	Glioblastoma cell lines	Replicated efficiently in tumor cell lines; less efficiently in cells contained decrease miR‐21 activity	Increased cell specificity	^202^
	AP27i145	miR‐145	Non‐small cell lung cancer cells	Engineered to express miRNA‐145; AP27i145 replication inversely correlated with the expression of miRNA‐145 in infected cells.	Improve selectivity	^203^
Poxviridae	VV‐miR‐34a	miR‐34a	Multiple Myeloma	Increase the release of cytochrome c that result in mitochondria‐initiated apoptosis	Enhance anticancer activity; Induce apoptosis; Decrease tumor growth	^204^
Picornaviridae	53a‐CVB	miR‐34a/c	Human non‐small cell Lung cancer cells	Inserting miR‐34a targets in both the 5′ UTR and 3′ UTR of the virus which is called the double‐miR‐34a targeting virus; Without toxic for normal tissue	Decrease the cytotoxicity	^173^

## 6 CONCLUSION

Because traditional cancer therapies such as chemotherapy, surgery, and even radiotherapy have limitations and have a number of negative side effects on patients, there is still a need for an effective treatment. In the search for novel effective anticancer therapies, virotherapy attracts attention due to its unique advantages, such as its lack of cross‐resistance with standard therapeutic agents and its great potential for tumor suppression through a different mechanism which can also specifically enter and be replicated within the tumor microenvironment.^174^ Despite the excellent and promising results of clinical trials using virotherapy agents in the treatment of various cancers, this novel therapy, like other therapeutic approaches, faced challenges. The challenges included the infectious nature of the virus, the determination of a delivery platform, an effective dose, and antiviral immunity.^175^ Currently, genetic engineering is used to eliminate the toxicity of viruses that are supposed to be used in cancer treatment and enhance their therapeutic effects.^176^ The combination of virotherapy agents with conventional therapies is another solution that results in enhanced anticancer effects without cross‐resistance and also allows using lower doses of the virus, resulting in a reduction in virus toxicity for normal cells.

Moreover, in the case of solid tumors with high mutational burdens which are also not easy to access, it is hard to achieve a cure with a single therapy. Therefore, combination therapy using virotherapy agents along with conventional therapies can improve the outcomes.^177^, ^178^, ^179^ In addition, genetic technology allows viruses to be used as a vector for the target delivery of anticancer therapeutic agents. These findings suggest the combination therapy of using a virotherapy agent along with conventional therapy is an excellent choice for treating the malignancy. However, further studies are needed in this field to develop the viruses as anticancer therapeutic agents in the future.

## AUTHOR CONTRIBUTIONS

**Hossein Javid:** Conceptualization (equal); project administration (equal); supervision (lead); writing – original draft (lead); writing – review and editing (lead). **Alireza Sharbaf Mashhad:** Investigation (equal); writing – original draft (equal); writing – review and editing (equal). **Shaghayegh Yazdani:** Investigation (equal); validation (equal); writing – original draft (equal); writing – review and editing (equal). **Mahsa Akbari Oryani:** Investigation (equal); writing – original draft (equal); writing – review and editing (equal). **Sanaz Akbari:** Investigation (equal); writing – original draft (equal); writing – review and editing (equal). **nastaran rezagholinejad:** Investigation (equal); validation (equal); writing – original draft (equal); writing – review and editing (equal). **Mahboubeh Tajaldini:** Writing – original draft (equal); writing – review and editing (equal). **Mehdi Karimi‐Shahri:** Investigation (lead); supervision (lead); validation (lead); writing – original draft (lead); writing – review and editing (lead).

## FUNDING INFORMATION

Funding information is not applicable/no funding was received.

## CONFLICT OF INTEREST STATEMENT

On behalf of all authors, the corresponding author states that there is no conflict of interest.

## ETHICS APPROVAL

Ethics approval for this type of article (A review) is not applicable.

## Data Availability

Data sharing is not applicable to this article as no datasets were generated or analyzed during the current study.
